# The Relationship between Carotid Artery Stenosis and the Severity of Fatty Liver in Patients with Coronary Artery Disease Who Are Candidates for Angiography

**DOI:** 10.34172/mejdd.2025.439

**Published:** 2025-10-31

**Authors:** Mehdi Pezeshgi Modarres, Ahmad Hormati, Mahdieh Memar, Alireza Shahhamzeh, Mostafa Vahedian, Mohammad Hosein Atarod

**Affiliations:** ^1^Center for Research on Disease, Hepatology and Gastroenterology, Qom University of Medical Sciences, Qom, Iran; ^2^Department of Internal Medicine, School of Medicine, Liver and Pancreatobiliary Diseases Research Center, Digestive Diseases Research Institute, Shariati Hospital, Tehran University of Medical Sciences, Tehran, Iran; ^3^School of Medicine, Qom University of Medical Sciences, Qom, Iran; ^4^Department of Family and Community Medicine, School of Medicine, Spiritual Health Research Center, Qom University of Medical Sciences, Qom, Iran; ^5^Student Research Committee, Faculty of Medicine, Qom University of Medical Sciences, Qom, Iran

**Keywords:** Non-alcoholic fatty liver disease (NAFLD), Carotid artery stenosis, Coronary artery disease, FIB-4 index, Ultrasound

## Abstract

**Background:**

Non-alcoholic fatty liver disease (NAFLD) is the most common liver disorder in developed societies, which can lead to inflammation, fibrosis, and even cirrhosis. Recent studies have also suggested an association between NAFLD and an increased risk of cardiovascular diseases through inflammatory mechanisms and insulin resistance. This study aimed to investigate the relationship between carotid artery stenosis and liver fibrosis severity in patients with coronary artery disease (CAD).

**Methods:**

In this cross-sectional study, 200 patients referred for coronary angiography at Shahid Beheshti hospital, Qom, Iran, in 2019 were evaluated. Based on carotid Doppler ultrasonographic findings, patients were divided into two groups: with and without carotid stenosis. The severity of fatty liver was assessed using ultrasound, and fibrosis was evaluated using the fibrosis-4 (FIB-4) index. Demographic, laboratory, and imaging data were analyzed using SPSS software.

**Results:**

Among 200 patients (mean age 58.04 years, 50.5% female), 50% had carotid stenosis. Patients with carotid stenosis had a grade II and III fatty liver (44% with grade 2-3 vs. 19% in the non-stenosis group, *P*=0.001) and a higher FIB-4 score (40% vs. 8%, *P*<0.001). Additionally, patients with high FIB-4 scores had more severe coronary artery involvement (62.5% with 2-3 vessel disease vs. 40.8%, *P*=0.003) and a higher prevalence of carotid stenosis (83.3% vs. 39.5%, *P*<0.001).

**Conclusion:**

This study demonstrated that liver fibrosis severity (based on the FIB-4 index) is significantly associated with carotid artery stenosis and coronary artery involvement. Therefore, assessing liver fibrosis may serve as a predictive marker for cardiovascular risk in patients with NAFLD. The use of non-invasive methods such as FIB-4 alongside ultrasonography is recommended for fibrosis evaluation.

## Introduction

 Non-alcoholic fatty liver disease (NAFLD) is the most common liver disease and one of the most prevalent physical disorders in developed societies, known to disrupt liver function.^1^ The prevalence of NAFLD in society is estimated to be 14%-23%, and this figure is higher in obese and patients with type 2 diabetes mellitus, reaching 70%-90%.^2^ Considering that NAFLD is a benign condition, it is usually asymptomatic and is detected incidentally in imaging studies or when investigating the cause of increased liver enzymes. This is why NAFLD has long been considered an incidental finding and has recently received increasing attention.^3^ Fortunately, most cases of fatty liver disease can be diagnosed through simple blood tests or by performing non-invasive imaging methods, such as liver sonography.^4^ Weight loss and regulation of blood fat levels in patients are effective in preventing the progression of liver damage.^5^ Hepatomegaly is present in 75% of patients, while advanced signs (spider angioma, ascites, splenomegaly, palmar erythema) are rare.^6^ In recent years, studies have been conducted on the relationship between fatty liver and atherosclerosis, and the results of these studies indicate the relationship between fatty liver and coronary artery atherosclerosis.^7^ Fatty liver increases the risk of cardiovascular diseases through classical mechanisms such as dyslipidemia, high blood pressure, and diabetes. If the classic risk factor is accompanied by fatty liver, it increases the risk of metabolic factors and aggravates coronary artery disease (CAD).^8,9^ An increase in the thickness of the intima-media layer of the carotid artery and stenosis of the common carotid artery secondary to carotid plaques are markers of atherosclerosis.^10,11^ While there is a strong association between fatty liver and cardiovascular risk factors, more research is needed to determine the exact relationship between the two. Fatty liver may contribute to an increased risk of heart disease through mechanisms such as inflammation, insulin resistance, and oxidative stress. However, it is also possible that the presence of other risk factors may be driving the increased risk of heart disease in patients with fatty liver. The lack of such a complete study is felt in the review of the studies that have been carried out. Therefore, we decided to conduct a study to investigate the relationship between carotid artery stenosis and fatty liver fibrosis, and fatty liver criteria in patients with ischemic heart disease (IHD), to reduce the risk of life and financial complications in these patients.

## Materials and Methods

###  Study Design and Setting

 This was a cross-sectional study conducted in 2019 at Shahid Beheshti Hospital, Qom, Iran. The study population comprised adult inpatients who were candidates for coronary angiography and had been referred to the angiography unit of Shahid Beheshti hospital. Convenience sampling was employed for participant recruitment.

###  Sample Size Calculation

 The sample size was calculated based on a previous similar study,^12^ considering an alpha (Type I error) of 0.05, a statistical power of 0.80, and an odds ratio (OR) of 1.70. This calculation indicated a minimum required sample size of 184 participants. To account for potential attrition or incomplete data, a total of 200 patients were initially enrolled in the study.

###  Inclusion and Exclusion Criteria

####  Inclusion Criteria

 Patients suspected of CAD, with a confirmed diagnosis of steatotic liver disease (SLD) by ultrasound, and with available liver function test results were eligible. All participants provided written informed consent before study enrollment.

####  Exclusion Criteria

 Patients with any of the following conditions were excluded:

- Did not provide informed consent.
- History of myocardial infarction, cerebrovascular accident, or congestive heart failure.
- Presence of congenital cerebral or coronary vascular anomalies.
- Genetic disorders associated with hypercholesterolemia.
- Diagnosed with chronic liver diseases such as hepatitis C, hepatitis B, Wilson’s disease, hemochromatosis, or autoimmune hepatitis.

###  Data Collection

 Following admission and CAD diagnosis via angiography, all eligible patients underwent comprehensive assessments:

1. Clinical and biochemical data: Blood samples were collected from all patients for routine biochemical tests, including liver enzymes (aspartate aminotransferase [AST] and alanine aminotransferase [ALT]), lipid profile (cholesterol, triglycerides, low-density lipoproteins [LDL], and high-density lipoproteins [HDL]), and platelet count. All laboratory analyses were performed at the central laboratory of Shahid Beheshti hospital.
2. FIB-4 Score Calculation: The Fibrosis-4 (FIB-4) index, a non-invasive marker for liver fibrosis severity, was calculated for each patient using their age, AST, ALT, and platelet count, according to the standard formula:

FIB-4 = Age * AST / (0.001 * Platelets * sqr(ALT))

 This score was incorporated as an additional measure to assess the severity of liver disease, aiming to enhance the validity and scientific power of our study regarding the relationship between carotid artery stenosis and SLD severity.

####  Ultrasound Examinations

- Liver ultrasound: B-mode liver ultrasound was performed for the diagnosis and grading of SLD based on standard qualitative criteria (e.g., mild/grade 1, moderate/grade 2, severe/grade 3).
- Carotid artery color Doppler ultrasound: Carotid artery color Doppler ultrasound was performed to assess the presence and degree of carotid artery obstruction.
- All ultrasound examinations (liver and carotid) were performed by a single experienced sonographer blinded to patients’ clinical, laboratory, and angiography findings to minimize diagnostic bias using a Samsung WS80 ultrasound machine to ensure consistency.

####  Patient Grouping

 Based on the results of the carotid artery color Doppler ultrasound, patients were divided into two main comparison groups, each consisting of 100 individuals: Group 1 comprised patients with carotid artery obstruction, and group 2 comprised patients with normal carotid arteries. The severity of SLD, liver enzyme levels, and FIB-4 scores were then evaluated and compared between these two groups.

###  Statistical Analysis

 Statistical analyses were performed using SPSS software (version 25.0, IBM Corp., Armonk, NY, USA). Continuous variables, including FIB-4 scores, were assessed for normality of distribution using appropriate tests (e.g., Shapiro-Wilk test). Depending on their distribution, independent samples t-tests or Mann-Whitney U tests were used to compare quantitative values (e.g., FIB-4 scores, liver enzymes) between the two groups. For comparisons involving more than two groups (if necessary for secondary analyses), analysis of variance (ANOVA) was employed. Categorical variables were analyzed using the chi-square test. A *P* value less than 0.05 was considered statistically significant.

## Results

 A total of 200 patients with suspected CAD were enrolled (mean age: 58.04 ± 11.93 years; 50.5% female). Mean body mass index (BMI) was 31.3 ± 1.41 kg/m^2^, and 77.5% had comorbidities. Age distribution and laboratory profiles are summarized in Table 1.

**Table 1:** Baseline characteristics and laboratory findings

**Variable**	**Mean/frequency**	**SD/Percent **
Age (years)		
Sex	58.04	11.93
Men	99	49.5
Women	101	50.5
Body mass index (BMI, kg/m^2^)	31.3	1.41
Comorbidities, n (%)		
Yes	155	77.5
No	45	22.5
Laboratory		
White blood cells (WBC, × 10³/µL)	7991	2685
Hemoglobin (Hb, g/dL)	14.17	1.35
Platelets ( × 10⁹/L)	241,841	67,176
Aspartate aminotransferase (AST, U/L)	31.66	16.08
Alanine aminotransferase (ALT, U/L)	31.10	21.26
Alkaline phosphatase (ALP, U/L)	185.14	60.9
International normalized ratio (INR)	1.05	0.08
Prothrombin time (PT, s)	12.63	0.77
Fasting blood sugar (FBS, mg/dL)	143.83	55.6

 The study population demonstrated significant metabolic and cardiovascular pathology, with liver ultrasound revealing grade 1 (mild) steatosis in 137 patients (68.5%) and grades 2-3 (moderate-severe) steatosis in 63 patients (31.5%). Fibrosis risk stratification using FIB-4 scores categorized patients as low risk ( < 1.3) in 82 cases (41.0%), indeterminate risk (1.3-2.67) in 70 cases (35.0%), and high risk ( > 2.67) in 48 cases (24.0%). Cardiovascular evaluation identified carotid stenosis in 100 patients (50.0%), while coronary angiography showed 0-vessel disease in 47 patients (23.5%), 1-vessel occlusion in 61 (30.5%), 2-vessel disease in 36 (18.0%), and 3-vessel involvement in 56 patients (28.0%).

 The findings between patients with positive and negative carotid stenosis revealed statistically significant differences in several key variables. Regarding ultrasound grading of fatty liver, only 56% of patients in the stenosis-positive group had grade 1, while this proportion reached 81% in the stenosis-negative group (*P* = 0.001). Additionally, the percentage of patients with high fibrosis risk (FIB-4 > 2.67) was significantly higher in the stenosis-positive group (40% vs. 8%, *P* < 0.001). Although there were no statistically significant differences in age and sex distribution between the two groups (*P* = 0.810 and *P* = 0.777, respectively), liver enzyme levels (alkaline phosphatase [ALP], ALT, and AST) were significantly higher in the stenosis-positive group (*P* < 0.001 for all). Interestingly, the BMI in the stenosis-positive group was slightly but significantly lower than in the stenosis-negative group (*P* < 0.001, Table 2).

**Table 2:** Carotid stenosis vs. liver disease parameters

**Variable**		**Stenosis (+) (n=100)**	**Stenosis (–) (n=100)**	***P *** **value**
Ultrasound grade of steatotic liver disease (SLD)	Grade 1	56 (56.0%)	81 (81.0%)	0.001
	Grades 2–3	44 (44.0%)	19 (19.0%)	
Fibrosis-4 (FIB-4) > 2.67		40 (40.0%)	8 (8.0%)	< 0.001
Age	30 -40	5 (5)	3 (3)	0.810
	40-50	23 (23)	30 (30)	
	50-60	31 (31)	26 (26)	
	60 – 70	23 (23)	25 (25)	
	80 - 80	12 (12)	12 (12)	
Sex	Male	51 (51)	48 (48)	0.777
	Female	49 (49)	52 (52)	
Underlying disease (comorbidities)	Yes	77 (77)	79 (79)	0.856
	No	23 (23)	21 (21)	
Alkaline phosphatase (ALP)	Normal	80 (80)	100 (100)	< 0.001
	Abnormal	20 (20)	0 (0)	
Alanine aminotransferase (ALT)	Normal	80 (80)	100 (100)	< 0.001
	Abnormal	20 (20)	0 (0)	
Aspartate aminotransferase (AST)	Normal	80 (80)	100 (100)	< 0.001
	Abnormal	20 (20)	0 (0)	
Body mass index (BMI, kg/m^2^)		31.26 ± 1.34	31.34 ± 1.48	< 0.001

 The results of Table 3 demonstrate a significant association between the severity of liver fibrosis (based on FIB-4 score) and vascular involvement. In patients with high fibrosis risk (FIB-4 > 2.67), the prevalence of carotid stenosis was significantly higher (83.3% vs. 39.5%, *P* < 0.001). Additionally, this group of patients showed more severe coronary artery involvement, with 62.5% having 2-3 vessel disease compared with only 40.8% in the low FIB-4 group (*P* = 0.003). In contrast, patients with lower FIB-4 scores predominantly had milder coronary artery involvement (26.3% with no vessel disease and 32.9% with single vessel disease).

**Table 3:** CAD severity by liver fibrosis risk

**Variable**		**FIB-4≤2.67 (n=152)**	**FIB-4>2.67 (n=48)**	***P*** ** value**
Vessel occlusion	0-vessel	40 (26.3%)	7 (14.6%)	0.003
	1-vessel	50 (32.9%)	11 (22.9%)	
	2–3-vessel	62 (40.8%)	30 (62.5%)	
Carotid stenosis		60 (39.5%)	40 (83.3%)	< 0.001

 The comparative analysis between patients with grade 1 versus grades 2-3 fatty liver revealed no statistically significant differences in demographic and clinical characteristics (all *P* values > 0.05). The age distribution showed similar patterns across decades, with the majority of patients in both groups falling within the 50-60-year range (29.2% grade 1 vs. 27% grades 2-3, *P* = 0.713). Sex distribution was balanced, with males representing 51.1% of grade 1 and 46% of grades 2-3 patients (*P* = 0.545). Comorbidity prevalence was comparable between groups (76.5% in grade 1 vs. 81% in grades 2-3, *P* = 0.856), as was mean BMI (31.23 ± 1.46 vs. 31.45 ± 1.29 kg/m^2^, *P* = 0.921, Table 4).

**Table 4:** Examining demographic and paraclinical information by separating the two groups in terms of liver sonography

**Variable**		**Liver sonography **		***P*** ** value**
		**Grade 1**	**Grade 2,3**	
Age	30 -40	6 (4.4)	2 (3.2)	0.713
	40-50	36 (26.3)	17 (27)	
	50-60	40 (29.2)	17 (27)	
	60-70	29 (21.2)	19 (30.2)	
	70-80	19 (13.9)	5 (7.9)	
	80-90	7 (5.1)	3 (4.8)	
Sex	Male	70 (51.1)	29 (46)	0.545
	Female	67 (48.9)	34 (54)	
Underlying disease	Yes	104 (76.5)	51 (81)	0.856
	No	32 (23.5)	12 (19)	
BMI		31.23 ± 1.46	31.45 ± 1.29	0.921

## Discussion

 This study aimed to determine the relationship between carotid artery stenosis and the severity of fatty liver in patients with CAD. 200 patients with an average age of 58.04 and a sex breakdown of 101 (50.5%) female patients were examined. The findings of liver ultrasound alone and liver ultrasound together with liver enzymes, separately for the two groups, showed a significant relationship between the two studied groups in the examination of these variables (*P* = 0.000).

 Our findings demonstrate that the FIB-4 index (as a non-invasive marker of fibrosis) is a stronger predictor of carotid stenosis than ultrasound-based steatosis grading. This confirms that liver fibrosis (not merely fat accumulation) is the primary driver of cardiovascular risk in Metabolic dysfunction–associated steatotic liver disease (MASLD).^11^ Wong and colleagues also emphasized the association between NAFLD and CAD, but by incorporating fibrosis assessment (FIB-4), we more precisely elucidated the underlying mechanism: systemic inflammation and oxidative stress secondary to liver fibrosis accelerate the pathogenesis of atherosclerosis.^12^

 In the end, the results of our study showed that fatty liver disease could play a role in increasing the thickness of the intima-media layer of the carotid artery as an indicator of atherosclerosis, which can be seen even in mild cases of fatty liver. In this regard, Wong and colleagues conducted a study in Hong Kong in 2011 to determine the role of fatty liver in predicting CAD and the clinical outcomes of patients undergoing angiography.^13^ In this prospective cohort study, 612 consecutive patients underwent coronary angiography with ultrasound screening for fatty liver. Finally, it was stated that while fatty liver cannot predict mortality from cardiovascular diseases in patients with CAD, there was a relationship between NAFLD and CAD in patients who had clinical manifestations of coronary angiography. Furthermore, the results of our study also indicated that the prevalence of fatty liver in patients who were included in the study with abnormal carotid ultrasound was significantly higher than in the group with normal carotid ultrasound. Also, Angel Brea and others stated that patients with NAFLD showed a set of risk factors for metabolic syndrome and advanced carotid atherosclerosis. NAFLD seems to be a feature of metabolic syndrome, and its detection in abdominal ultrasound should warn of an increased cardiovascular risk,^1^ which was consistent with the results of our study.

 In another study, Fakharian et al^12^ conducted a case-control study in Birjand in 2015 to evaluate the effect of NAFLD on the thickness of the intima-media of the carotid artery as a risk factor for atherosclerosis. They concluded that fatty liver disease could play a role in increasing the thickness of the intima media layer of the carotid artery as an indicator of atherosclerosis, which can be seen even in mild degrees of fatty liver. The results of our study also showed that fatty liver affects the function of coronary arteries and can disrupt the function of these vessels. The results of Fakharian study were also consistent with our study.^12^ In another study in 2012 in Isfahan, adibi and colleagues conducted a study to investigate the relationship between NAFLD and CAD in patients with angina pectoris.^14^ The results of their study showed that NAFLD was a risk factor for CAD in patients with angina pectoris. Also, the increase in the prevalence of NAFLD could lead to an increase in cardiovascular diseases. The results of our study also confirmed this issue and showed that fatty liver can be a risk factor for patients with CAD. Other studies, similar to Silvia Sookoian’s, aimed to investigate the relationship between severe NAFLD and carotid atherosclerosis.^15^ They concluded that fatty liver disease can play a role in increasing the thickness of the intima-media layer of the carotid artery as an indicator of atherosclerosis. This problem can be seen even in mild degrees of fatty liver.

 Traditional fatty liver assessment by ultrasonography is insufficient for predicting cardiovascular complications. Our findings demonstrated that the FIB-4 index (as a fibrosis marker) showed a stronger association with carotid stenosis. Therefore, in patients with MASLD, we recommend complementing ultrasonography with non-invasive fibrosis evaluation tools such as FIB-4 or two-dimensional shear wave elastography (2D-SWE) for cardiovascular risk stratification.

## limitation

 The most significant limitation was the use of ultrasonography as the sole method for fatty liver assessment, which cannot evaluate fibrosis. Although FIB-4 partially compensated for this weakness, the use of more advanced techniques, such as 2D-SWE is recommended for future studies.^16^

## Conclusion

 The results of our study showed that there was a higher prevalence of carotid artery stenosis in patients with fatty liver. Additionally, the severity of fatty liver is directly correlated with coronary artery involvement in patients with CAD who are undergoing angiography.
